# Epileptic Seizure Prediction Based on Hybrid Seek Optimization Tuned Ensemble Classifier Using EEG Signals

**DOI:** 10.3390/s23010423

**Published:** 2022-12-30

**Authors:** Bhaskar Kapoor, Bharti Nagpal, Praphula Kumar Jain, Ajith Abraham, Lubna Abdelkareim Gabralla

**Affiliations:** 1 Ambedkar Institute of Advanced Communication Technologies & Research (AIACT&R), Guru Gobind Singh Indraprastha University, New Delhi 110078, India; 2 NSUT (East Campus) (Formerly AIACT&R), Delhi 110031, India; 3 Department of Computer Engineering & Applications, GLA University, Mathura 281406, India; 4 Machine Intelligence Research Labs (MIR Labs), Auburn, WA 98071, USA; 5 Department of Computer Science and Information Technology, College of Applied, Princess Nourah bint Abdulrahman University, Riyadh 11564, Saudi Arabia

**Keywords:** electroencephalograph (EEG), epileptic seizure prediction, ensemble classifier, corvid and gregarious search agents, hybrid seek optimization

## Abstract

Visual analysis of an electroencephalogram (EEG) by medical professionals is highly time-consuming and the information is difficult to process. To overcome these limitations, several automated seizure detection strategies have been introduced by combining signal processing and machine learning. This paper proposes a hybrid optimization-controlled ensemble classifier comprising the AdaBoost classifier, random forest (RF) classifier, and the decision tree (DT) classifier for the automatic analysis of an EEG signal dataset to predict an epileptic seizure. The EEG signal is pre-processed initially to make it suitable for feature selection. The feature selection process receives the alpha, beta, delta, theta, and gamma wave data from the EEG, where the significant features, such as statistical features, wavelet features, and entropy-based features, are extracted by the proposed hybrid seek optimization algorithm. These extracted features are fed forward to the proposed ensemble classifier that produces the predicted output. By the combination of corvid and gregarious search agent characteristics, the proposed hybrid seek optimization technique has been developed, and is used to evaluate the fusion parameters of the ensemble classifier. The suggested technique’s accuracy, sensitivity, and specificity are determined to be 96.6120%, 94.6736%, and 91.3684%, respectively, for the CHB-MIT database. This demonstrates the effectiveness of the suggested technique for early seizure prediction. The accuracy, sensitivity, and specificity of the proposed technique are 95.3090%, 93.1766%, and 90.0654%, respectively, for the Siena Scalp database, again demonstrating its efficacy in the early seizure prediction process.

## 1. Introduction

The human brain is a key component of the central nervous system (CNS), and epilepsy is a common neurological condition that affects the CNS in the brain. According to a report by the International League Against Epilepsy (ILAE) [1], epilepsy is a neurological brain disorder that occurs due to the symptoms of an epileptic seizure. A sudden, uncontrolled, electrical disturbance in the brain is known as a seizure. It can change your emotions, actions, behavior, and degree of consciousness. Epilepsy is typically defined as having two or more seizures that are unprovoked and occur at least 24 h apart [2]. A person with epilepsy may experience a single seizure or a variety of them. Generalized, focal, and unidentified seizures are the three primary types of seizures. *Focal seizures*, also known as a partial seizure, occur in about 60% of people with epilepsy. The characteristics of a focused seizure can occasionally be confused with indications of mental illness or other types of neurological conditions. A person may experience both motor and non-motor symptoms as the seizure intensifies [3]. *Generalized seizures* can also be either non-motor, which does not entail physical movement, or motor, which entails physical movements such as jerking motions, limp or weak limbs, rigid muscles, muscle twitching, or full-body epileptic spasms [4]. *Tonic-clonic seizures* or *grand-mal seizures* are another form of motor seizures which include stiffening, loss of consciousness, rhythmic jerking, bluish skin from oxygen deprivation, and/or loss of bladder and/or bowel control [5].

Abnormal activities of the brain, such as loss of consciousness, sensation, or other cognitive functions, are the causes of epileptic seizures [6]. A recent analysis shows that about 23 to 100 per 100,000 people are affected with epilepsy. People of extreme age are more likely to be affected, and it peaks in people between 10 and 20 years old. Around 70 million people worldwide, or 1% of the population, suffer from epilepsy. Despite taking numerous anti-seizure drugs, 30–40% of people living with epilepsy still experience seizures [7]. In severe cases such as focal epilepsy, patients are subjected to surgical procedures, but it is not advisable to take such measures for about 30% of the patients. Hence, once a seizure is identified, it is important to control the consequent seizures [8]. It is possible to record the abnormal activity of the brain before the occurrence of a seizure with electroencephalogram (EEG) signals [9,10]. EEG is a prominent tool for analyzing epilepsy by recording the activities of the human brain. EEG is mostly used for brain-related diseases as it is non-invasive, highly accurate, and economical. According to the length of the episode, epileptic seizures are divided into a variety of stages. The ictal state refers to the state that initiates with an onset and ends with an epileptic seizure. The postictal period begins after the seizure has ended and lasts for a short while. The preictal state begins around 60 to 90 min before the commencement of the seizure and is referred to as common brain activity or interictal state. The scanning of seizures visually is time-consuming, particularly when the EEG signal is very long. In such cases, automatic computer-based diagnosis is preferential, where the features of the EEG signals are used for diagnosis [11]. In addition to being extremely expensive for the person with epilepsy, their family, and society as a whole, seizures are difficult to control. Seizures that go unchecked significantly lower a person’s quality of life. In terms of the global load of disease, which depends on years lost by individuals due to premature mortality and years spent in less-than-optimal health, epilepsy represents more than 0.5% of the total. Epilepsy has significant financial repercussions regarding medical expenses, preventable deaths, and missed productivity at work. An Indian cost-effectiveness study found that public support for first- and second-line therapies and other medical costs helped lessen the financial burden that epilepsy creates. Since epilepsy is stigmatized and discriminated against globally, even if social effects vary from country to country, epilepsy is occasionally more challenging to manage than the seizures themselves. Epilepsy sufferers may be the object of prejudice. People who want to avoid being associated with the condition may be discouraged from getting treatment due to stigma. By 2050, the number of people over 65 is predicted to rise from 461 million to 2 billion. The social and medical effects of this large growth will be profound. HAR is developing as a potent tool for monitoring older individuals’ physical, functional, and cognitive health in their homes [12].

Early trials of automatic diagnosis resulted in systems with an accuracy of 76% to 90% and a false detection rate of 1 to 0.71/h. Hence, it is necessary to develop computationally effective methods. EEG recordings contain a huge amount of data; thus, technologies are required to be developed to handle the classification and feature selection processes. Some challenges in seizure prediction methods are listed below:

1. One of the primary drawbacks of generative adversarial networks LSTM unit is the right or left amplitude predominance in EEG readings;
2. Methods for predicting epileptic seizures based on the support vector machine and K-nearest neighbors inherit the problem of lacking directionality and phase-related data;
3. The EEG spike rate technique-based seizure prediction algorithm does not employ deep learning methodology. Consequently, utilizing this method does not allow for the accurate evaluation of an epileptic episode;
4. The major problem associated with the generative adversarial networks strategy for the prediction of seizure is that it is unsuccessful in enhancing the anticipation time;
5. Deep learning algorithms for seizure prediction are hampered by lower SNR and a higher number of parameter inputs.

The problem of reduced classification accuracy in seizure prediction methods has been addressed by several researchers. Most of the seizure prediction strategies are user-specific due to the variation in the type and location of the seizure with the EEG signals of patients. The conventional technique of seizure prediction consists of processes such as pre-processing of signals, selection of features, and classification [13,14,15,16]. The pre-processing step is executed to remove unwanted noise, enhance signal quality, and so on. Pre-processing is carried out with band pass/band stop filtering, Fourier transforms (FT), empirical mode decomposition, wavelet transform (WT), and Hilbert vibration decomposition. The methods that do not follow the initial pre-processing are suspected of possessing reduced specificity and sensitivity. Following this step, the signal goes through a feature selection method to extract the informative characteristics. If the size of the feature is large, its dimensions can be reduced to form a feature vector. The classification component performs the final step, where the feature vector is tested to find the best approach to categorize the characteristics based on the hidden pattern.

The major goal of this research concentrates on developing an optimized seizure prediction method using a hybrid seek optimization-based ensemble classifier. The EEG signal acts as the input for the classification module, where all the waves (i.e., alpha, beta, delta, theta, and gamma) of the EEG signals are subjected to a feature selection process. The EEG signals are further processed to extract statistical, wavelet, and entropy-based features [17] for reducing the prediction complexity. Finally, the significant features are analyzed with the proposed hybrid seek optimization-based ensemble classifier for seizure prediction. The optimization technique, named hybrid seek optimization, is based on the corvid and the gregarious search agents. The classifiers, such as AdaBoost, random forest, and decision tree, are combined as an ensemble classifier in the proposed seizure prediction module. The ensemble classifier is finely tuned in such a way as to produce the prediction output with enhanced accuracy. This paper suggests an intelligent seizure prediction module based on a hybrid seek optimization-based ensemble classifier employed for predicting seizure disease utilizing the EEG signal of the patients. To effectively achieve this outcome, the following contributions are made:

1. This research proposed a hybrid seek optimization-based ensemble classifier for seizure prediction with EEG signals. Advanced feature selection techniques have been used with EEG to improve findings and for simplification. This paper presents an innovative seizure prediction paradigm to provide researchers with a benchmark;
2. With the hybrid characteristics of gregarious and corvid search agents, a unique hybrid seek optimization method is created to make it easier for the ensemble classifier’s hyper-parameters to function;
3. Experiments have been conducted with a python tool installed in Windows 10 OS on CHB-MIT and Siena Scalp EEG databases. The outcomes demonstrate that the suggested model outperformed both datasets without experiencing over- or under-fitting issues;
4. Based on performance indicators utilizing the CHB-MIT database and Siena database in terms of training % and k-fold value, the comparative study revealed the viability of a hybrid seek optimization-based ensemble classifier for the seizure prediction module;
5. Compared to other techniques, it is clear that the hybrid seek-based ensemble classifier may provide enhanced seizure prediction while achieving higher accuracy, sensitivity, and specificity levels.

The sections of this research are structured as follows: Section 2 presents the literature based on the current seizure prediction models and their drawbacks. Section 3 explains the fusion parameter estimation for the strategy for seizure prediction. Section 4 demonstrates the outcomes along with a performance analysis of the proposed seizure prediction system, and finally, the paper is concluded with Section 5.

## 2. Related Work

**Figure 1:**
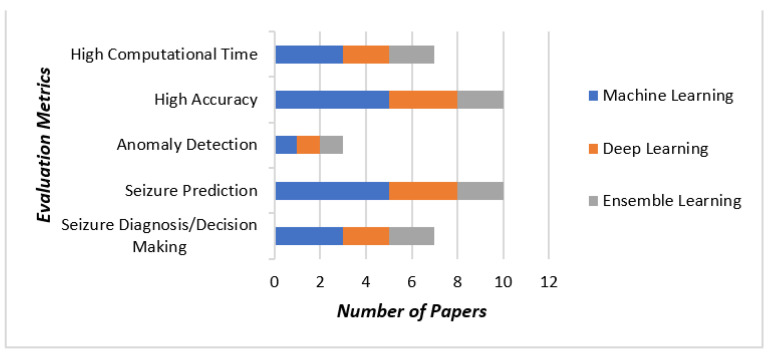
Outline of the distribution of three methods concerning evaluation metrics considered in this review.

There are many research contributions to seizure disease prediction techniques in the medical field literature and the most recent are summarized in this section. Khakon Das et al. [18] developed a model for identifying epileptic seizure waveforms from the pre-ictal phase of the EEG signal. This method detects the seizure at its initial stage and produces an alarm to make the neurologists aware of the condition. However, occasionally, the occurrence of left or right amplitude preponderance was noted and is considered the major drawback of this method. Marzieh Savadkoohi et al. [19] used classifiers, such as K-nearest neighbors (KNN) and support vector machine (SVM), for the prediction of a seizure, which was efficient, reliable, and flexible, and thus could be used for any range of frequency variation. However, it possessed poor phase information and directionality. Itaf Ben Slimen et al. [20] introduced a seizure detection approach based on spike rate, which was highly accurate and helped improve epileptic disease patients’ quality of life. However, a deep learning strategy was not used in the prediction process; thus, this may lead to performance degradation. Syed Muhammad Usman et al. [21] developed the generative adversarial networks long short term memory (LSTM) units with an enhanced sensitivity and reduced false positive alarm rate, but did not efficiently improve the anticipation time. Syed Muhammad Usman et al. [22] designed a prediction module for seizures using deep learning methods, obtaining an increased sensitivity and specificity. However, reduced SNR measures and the use of a large number of parameters were considered to be major limitations of this method. Chien-Liang Liu et al. [23] introduced a prediction model with a convolutional neural network framework that attained a shared indication of time and frequency domain features, but it could not be used to test the brain computer interface dataset. Heba M. Emara et al. [24] developed an anomaly detection strategy for multi-channel EEG signals, which was capable of attaining a high rate of prediction, but the need for a sample was the major drawback of this method. Hisham Daoud and Magdy A. Bayoumi [25] modeled the deep learning-based algorithms without the need to pre-process the input signal. This method completed the prediction process in less time with a reduced false alarm rate; however, this result mode was not capable of low variance entropy. Khakon Das et al. [26] implemented a seizure waveform for the detection of epileptic seizures. This model predicted an epileptic seizure in advance, and it is attracting significant attention in neuroscience. This model has a very high complexity and uses more computational time. Gang Wang et al. [27] executed an algorithm based on CNN and DTF, which is an accurate seizure prediction method that can be applied in a clinical setting and has advantages for the epilepsy patient as it is a closed-loop treatment; however, it was highly time-consuming. Syed Muhammad Usman et al. [28] devised an ensemble learning method for epileptic prediction with raised sensitivity without combining heart rate variability with EEG recordings in implementation. Many articles implement machine learning in healthcare Figure 1 shows the distribution of three methods of evaluation metrics considered in this literature review.

In the methods mentioned above, most of them suffered from disadvantages such as lack of data which is overcome in this research by the use of a standard dataset. Additionally, the procedures did not apply optimization algorithms to obtain an optimal solution. However, in this research, hybrid seek optimization is used to optimize the parameters. Most above-mentioned methods were computationally complex and consumed more time, but the proposed method reduced the time consumption of the classifier by optimal tuning.

## 3. Development of the Proposed Strategy

Information on the status of the brain is gathered by EEG signals, which are broadly utilized to analyze the different activities of the brain. In particular, they present significant data relevant to epileptic seizure disease. Epilepsy is a disease caused by a neurological disorder connecting disturbances in the nervous system induced by damage in the brain. It has been reported that about 1% of the world’s population is affected by a seizure disease.

**Figure 2:**
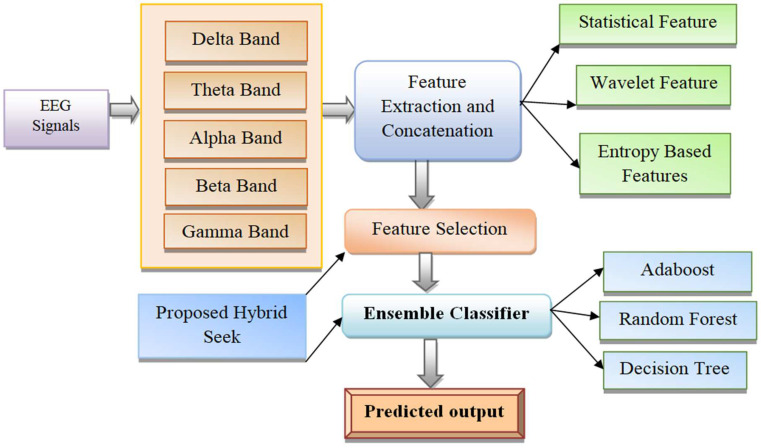
Proposed ensemble classifier-based seizure prediction module.

Visual analyses of EEG signals are tedious and time consuming, with lengthy EEG signals leading to increased error in measurements. Hence, artificial intelligence-based seizure prediction technologies are proposed to improve detection accuracy. Ensemble classifier-based EEG signal classification has attained enhanced attention from both industry and academia. This research explores using a new ensemble classifier to predict an epileptic seizure with noisy EEG signals. The schematic representation of the proposed model of seizure prediction is shown in Figure 2.

### 3.1. Pre-Processing of EEG Signals

**Figure 3:**
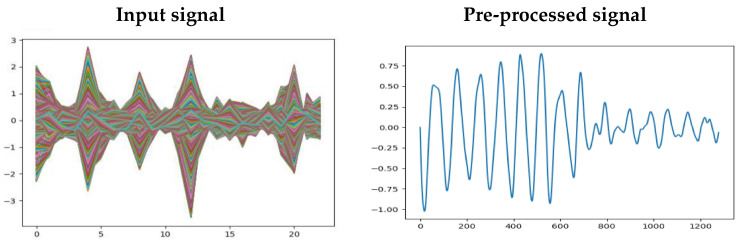
Pre-processed signals with artifacts and after elimination of input signals.

The first step of the proposed seizure detection module is pre-processing, intending to eliminate the artifacts present in the raw EEG signals containing nonlinear and non-stationary components. The artifacts are required to be pre-processed in such a way as to enhance the prediction accuracy of the proposed ensemble classifier. After the raw EEG signals have been downsampled, a band-pass filter is employed to exclude the frequencies that go beyond the proposed frequency threshold. The signals within the frequency range between 0 Hz and 75 Hz was used in this research, and the rest were eliminated to obtain a smooth EEG signal suitable for further processes. The pre-processed signal is shown in Figure 3.

### 3.2. Frequency Bands of EEG Signal

The production of frequency bands for the input EEG signal, such as alpha, beta, gamma, theta, and delta, are involved in the evaluation of inner-nodal data, enhancing recognition accuracy. The frequency of the delta band is between 0 and 4 Hz and the frequency of the theta band lies between 4 Hz and 8 Hz. The frequency of the alpha band is between 8 Hz and 13 Hz and the frequency of the beta band is between 13 Hz and 22 Hz. The frequency range of the gamma band varies at the higher frequency range of 22–30 Hz.

### 3.3. Feature Selection

Extracting significant features from each of the five frequency bands represents the next important step in the proposed seizure prediction module. The feature selection approach determines a feature vector from a regular vector represented in table1. A feature is a distinctive measurement that is extracted from a segment of a pattern of frequency bands in the proposed prediction module. It involves selecting the features or data that are the most significant to execute the classification process. The important features needed to be extracted in the proposed system are the statistical, wavelet, and entropy-based features. All the extracted feature and vectors are represented in Table 1.

#### 3.3.1. Statistical Features

These EEG signal features are used in the proposed classification approach to consider even minor variations in the original EEG signal. The statistical features are extracted from all five frequency bands and are described as:

(a) Mean: Mean is one of the most important statistical features which is used to evaluate the average of the total instances of the EEG signal to the total instances, expressed as:

(1) $$F_{MN}=\frac{1}{q}{\sum_{r=1}^{q}A}_{r}$$

where q represents the total instances of the EEG signal and $A_{r}$ represents the average of the features obtained from the $r^{th}$ data in the range [1,q].

(b) Variance: Variance is defined as the average of deviations in the square over the individual data, and the mean $F_{MN}$ is expressed as:

(2) $$F_{VR}=\frac{1}{q-1}\sum_{r=1}^{q}{\left(A_{r}-F_{MN}\right)}^{2}$$

Even a small variation in the measure of variance needs to be considered, as it may enhance the prediction performance.

(c) Standard deviation: The standard deviation is the assessment of widely dispersed data that determines the mean for each instance of the EEG signal and is considered as the root of the variance. It is represented by the equation:

(3) $$F_{SDN}=\sqrt{\frac{1}{q-1}\sum_{r=1}^{q}{\left(A_{r}-F_{MN}\right)}^{2}}$$

(d) Skewness: Skewness is defined as the assessment of asymmetry in relation to the third central moment’s rate. The normal division resembles the skewness to zero, and an entire symmetry database may have a zero skewness and is mathematically expressed as follows:

(4) $$F_{SKW}=\sum \frac{{\left(A_{r}-F_{MN}\right)}^{3}}{qd^{3}}$$

(e) Kurtosis: Kurtosis is measured by determining the value of the shared weight of the tails analogous to the enduring distribution that residue zero for Gaussian distribution. The expression for kurtosis is specified as,

(5) $$F_{KRT}=\sum \frac{{\left(A_{r}-F_{MN}\right)}^{4}}{qd^{4}}$$

where, $A_{r}$ indicates the $r^{th}$ value of A, and d is the sample standard deviation.

#### 3.3.2. Wavelet Features

The wavelet transform is suggested for its near-optimal time-frequency localization, multi-rate filtering, and multi-scale zooming features for the detection of transients in the system. The two important types of wavelet features are stated below:

(a) Wavelet energy: The energy after the decomposition of the wavelet sub-band is known as wavelet energy and is formulated as:

(6) $$B(w)={\sum_{r}\left|S_{w}\left|r\right|\right|}^{2}$$

where w is the level of decomposition and $S_{w}\left|r\right|$ is the factor of wavelet coefficient at the level r. The relative wavelet energy is evaluated with the ratio of normalized wavelet energy to the entire wavelet energy and is formulated as:

(7) $$F_{WEY}=\frac{B(w)}{\sum_{g_{co}=1}^{H}b(g_{co})}$$

(b) Wavelet entropy: In general, entropy is a measure of asymmetric, improbability, and disturbing signals. Uncertainties highly rely on the states and probability of the EEG signals. The value of wavelet entropy is generated as:

(8) $$F_{WEN}=\frac{B(w)}{\sum_{g_{co}=1}^{H}b(g_{co})}\log\frac{B(w)}{\sum_{g_{co}=1}^{H}b(g_{co})}$$

where $g_{co}$ represents the number of wavelet decompositions, b(g) denotes the wavelet coefficients, and the value of g is in the range [1, H].

#### 3.3.3. Entropy-Based Features

Entropy is an important measure of information due to its ability to quantify random variables’ improbability. It can scale the rate of information applicability effectively and can be applied in different domains. Redundancy, independence, and interdependence between the numbers of features are distinguished using the entropy-based measure. The entropy measure can be mathematically formulated as:

(9) $$F_{HEN}={\sum_{x=1}^{z\left(g\right)}L}_{x}\log L_{x}$$

where g indicates the attribute vector, z(g) indicates the number of unique values in g, and $L_{x}$ is the probability of $x^{th}$ information.

### 3.4. Feature Selection Using the Proposed Hybrid Seek Optimization

The feature vector is derived from the EEG input that contains the important patient data that will be examined. The feature vector, which may be written as feature vector, F, is made up of characteristics such as mean, variance, standard deviation, skewness, kurtosis, wavelet energy, wavelet entropy, and holo-entropy-based features.

(10) $$F=\left\{F_{MN},F_{VR},F_{SDN}.F_{SKW},F_{KRT},F_{WEY},F_{WEN},F_{HEN}\right\}$$

Finally, the feature vectors’ dimension is for predicting the EEG signals containing indications of a seizure. The solution encoding represents the statistical features as mentioned in Equation (10), and the optimization selects the best features to support the improved classification accuracy. The features of the EEG signal are represented in the feature vector. The selection of the significant features among all features plays an important role in the enhancement of accuracy in the proposed prediction module. Hence, significant features need to be selected using the proposed algorithm, which inherits the characteristics features of corvid and the gregarious search agents. The working principle of the proposed hybrid seek optimization algorithm is explained in the next section.

### 3.5. Proposed Hybrid Seek-Based Ensemble Classifier for Epileptic Seizure Prediction

In most experiments related to the prediction of a seizure disease, the classification is executed by a single classifier. In recent times, the successful use of ensemble classifiers, which are developed from individual classifiers, has motivated us to enhance the system effectiveness using multiple classifiers. The important benefit of such an ensemble classifier is that a collection of classifiers of similar characteristics is likely to provide enhanced performance compared to any of the classifiers on its own. The ensemble classifier in the proposed seizure prediction module comprises the combined characteristics of the classifiers such as the AdaBoost classifier, the random forest classifier, and the decision tree classifier that accepts the selected features of the EEG signals by the proposed hybrid seek optimization model to predict a seizure disease.

**Figure 4:**
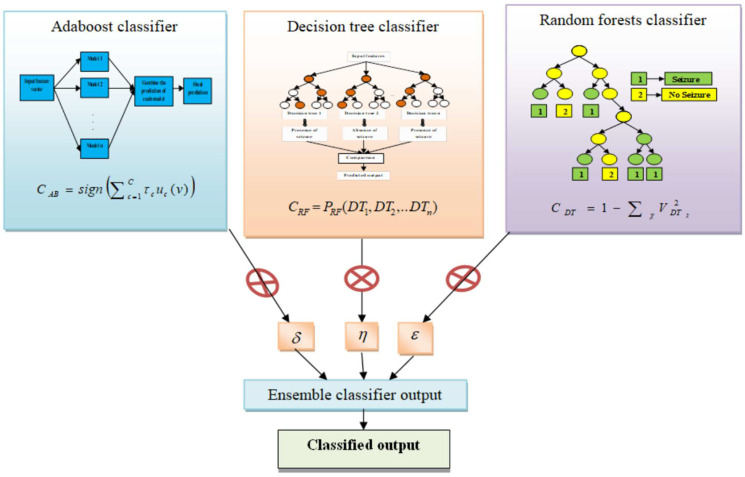
Proposed ensemble classifier for seizure prediction.

The classifiers in the proposed ensemble classifier are discussed in detail in this section to understand each classifier’s operating principle. The ensemble classifier has been developed using the Adaboost classifier, RF classifier, and the DT classifier in a way that the outputs from the individual classifiers are fused to represent the ensemble classifier’s output. The fusion parameters, i.e., δ,η, and ε merge the outputs from the individual classifiers of the ensemble classifier in such a way as to predict the presence or absence of seizure disease, with a condition of τ+ρ+ε=1. The output from the proposed ensemble classifier is represented as:

(11) $$E_{c}^{}=\tau E_{AB}+\rho E_{RF}+\varepsilon E_{DT}$$

where, τ,ρ, and ε are the individual outputs of the AdaBoost classifier, RF classifier, and the DT classifier, respectively, that are combined with the fusion parameters. A detailed analysis of the classifiers used in the proposed ensemble classifier is described below. The proposed ensemble classifier in seizure classification is depicted in Figure 4.

#### 3.5.1. AdaBoost Classifier

The AdaBoost algorithm is an iterative model that trains and assembles the weak classifiers into a strong classifier in such a way to obtain enhanced classification accuracy. The algorithm initially consigns a similar weight to the entire training set samples. A weak classifier, $u_{c}$, is then called for the classification of the samples, and the equivalent rate of classification error, $\varphi_{c}$, is evaluated. The term $\varphi_{c}$ involves updating each sample’s weight and evaluating the weight, $\tau_{c}$, of the weak classifier, $u_{c}$, in the subsequent iteration, and the processes are repeated. In the final step, the strong classifier, $U_{C}$, is accumulated from the weak classifiers and their equivalent weights. The error rate of classification by the weak classifier is expressed as:

(12) $$\varphi_{c}=\sum_{s=1}^{N}p_{s}^{c}E(u_{c}(v_{s})\neq t_{s})$$

where $u_{c}(v_{s})$ is the rate of prediction of the weak classifier, $t_{s}$ represents the true label, and E indicates the optimization function of the weight coefficient. The value s is in the range [1,N]. The term $p_{s}^{c}$ represents the weight measure of the present weak classifier. The weights of the weak classifiers that are grouped as strong classifiers are expressed as:

(13) $$\tau_{c}=\frac{1}{2}\ln\left(\frac{1-\varphi_{c}}{\varphi_{c}}\right)$$

**Figure 5:**
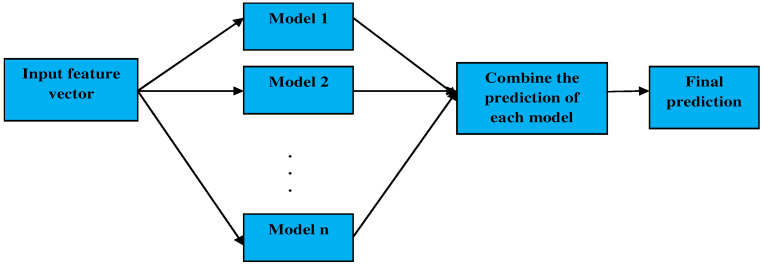
The architecture of the AdaBoost classifier.

With the combination of the weak classifiers and their optimized weights, the strong classifier can be obtained as:

(14) $$C_{AB}=sign\left(\sum_{c=1}^{C}\tau_{c}u_{c}(v)\right)$$

where C represents the count of weak classifiers and $C_{AB}$ is the prediction outcome of each weak classifier. The schematic representation of the AdaBoost classifier is shown in Figure 5.

#### 3.5.2. Random Forest Classifier

The RF classifier is a combination of the number of decision trees and hence individually acts as an ensemble learning-based algorithm. Each tree acts as a separate classifier, and the decision trees choose the classification outcome. The significant benefits of using an RF classifier are the increased accuracy in classification with resistance to overtraining, the capability to work with data sets of larger size, no need for the normalized features, and the need for only a few parameters in optimizations. These benefits are of particular concern when applied in early seizure detection. To develop an RF classifier consisting of R trees, the rules are as follows:

Step 1: Initially, the Q number of samples is obtained from the dataset, and it must be noted that all the training data may not be utilized, and only some data may be considered more than one time, while some may never be considered.

Step 2: If the dimension of the feature is D, then h is the dimension of the sub-features with the condition that h<D from the actual feature vectors. Then, h feature variables are chosen at random from the D features, and the best split is used to split the node.

**Figure 6:**
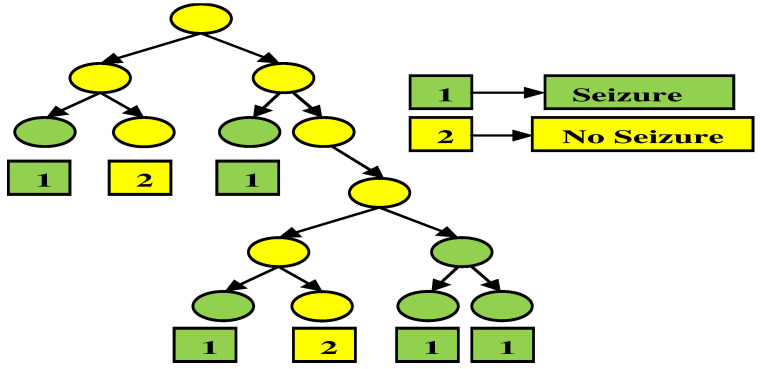
The architecture of the random forest classifier.

Step 3: Each tree keeps emerging until the entire training samples are completely divided without pruning, and the result thus obtained is represented as $C_{RF}$. As shown, the forest error rate relies on two factors: the reduced correlation between any two trees and the increased strength of the trees. The dimension reduction, h, significantly reduces the correlation and strength, so a trade-off between strength and correlation is necessary. The architecture of the random forest classifier is depicted in Figure 6.

#### 3.5.3. Decision Tree Classifier

**Figure 7:**
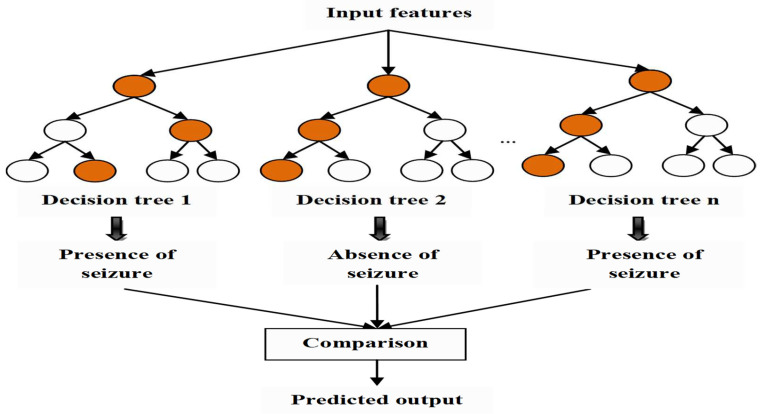
The architecture of the decision tree classifier.

DT is a classifier with a tree data construction comprising decision nodes and leaves. A leaf represents the classification, and the decision node indicates the test to be executed to appraise a single attributes. A solution is attained for the entire possible outputs of the analysis concerning a child node. The response of the decision tree to a series of samples is known as accuracy in classification. In other words, accuracy is defined as the part of rightly classified occurrences. A DT is concluded optimal with the classification of the dataset with increased precision and the existence of a few nodes. The local greedy search model is normally used in the DT to split the classes by assuming the information gain as the target function, and is formulated as:

(15) $$C_{DT}=1-\sum_{\chi }V_{DT_{\chi }}^{2}$$

where $V_{DT_{\chi }}^{}$ is the probability of the $\chi^{th}$ class. The layout for the decision tree is depicted in Figure 7.

### 3.6. Proposed Hybrid Seek Optimization in Fusion Parameter Estimation

The proposed hybrid seek optimization technique involves feature selection and identifies the ensemble classifier’s fusion parameters τ,ρ, and ε. The solution encoding represents the best set of fusion values within [0, 1] to support higher accuracy. The proposed algorithm uses gregarious and corvid search agents’ characteristics to resolve the feature selection and hyper parameter tuning optimization problems. The suggested algorithm assists by preserving a better relationship between the phases, such as exploration and exploitation, to generate improved outcomes in terms of both the local optimum solution and the ideal global solution. Both search agents pursue a seeking process to update the optimal position that is applied in the optimization process to find the solution to real-world optimization problems.

#### Proposed Hybrid Seek Optimization Algorithm

It is widely acknowledged that swarm intelligence (SI)-based optimization algorithms have been the main method for resolving global optimization issues because of their adaptability, simplicity, and improved effectiveness. In addition, the SI-based strategies mainly initiate randomness during the search process, apart from deterministic strategies. Using these strategies to attain the optimal global solution without getting trapped in the local optimal solution is of real significance. The corvid search agents are intelligent agents containing a giant brain irrespective of their size. They possess enhanced self-awareness and the ability to make tools. They remember faces and can retain information regarding the location of food even after several months. The characteristics of the corvid search agents are creating flocks, memorizing the positions of hidden food, following each other to steal the food, and protecting their young ones. These steps are discussed below,

Step 1: Population initialization: The optimization problem, decision parameters, and constraints are initialized in the population initialization step. The size of the flock of corvid search agents, $G_{size}$, the flight length, and the awareness probability, $P_{aw}$, is also initialized.

Step 2: Initialization of memory and position: The position of each corvid search agent, $J^{e}$, of the flock is initialized, with the condition e=1,2,…,m. The corvid search agents are positioned randomly. The memory of the corvid search agents is initialized as $Z_{i}^{G}$. At the initial stage, the corvid search agent possesses zero memory; hence, the food is assumed to be placed at the initial position.

Step 3: Evaluation of fitness solution: For each corvid search agent, the quality of the position is evaluated with the substitution of the decision variables in the objective function in such a way as to find the fitness measure in terms of accuracy.

Step 4: Generation of a new position: Consider if the corvid search agent e wants to generate a new position in the search space; it follows a new corvid search agent, f, of a randomly selected flock of corvid search agents. The new position of the corvid search agent e is formulated as:

(16) $$J_{CS}^{e,n+1}=J_{CS}^{e,n}+\lambda_{1}\times a^{e,n}\times \left(Z^{f,n}-J_{CS}^{e,n}\right)$$

where n is the iteration count, $\lambda_{1}$ is the random value and varies between 0 and 1, and a represents the flight length. The possibility of the corvid search agents getting trapped in the optimal local solution makes the solution non-preferable. In addition, the reduced searching precision of the corvid search agents needs to be enhanced; therefore, the characteristics of the gregarious search agents are introduced into the proposed system of optimization. The gregarious search agents are selected due to their flexible applications in practical engineering fields. The anti-predation characteristics of the gregarious search agents are the most important feature inherited with the corvid search agent. The gregarious search agent position is updated as:

(17) $$J_{GS}^{e,n+1}=\left\{\begin{array}{l} J_{best}^{n}+\theta \left|J_{GS}^{e,n}-J_{best}^{n,\partial }\;\right|,\;\;\;\;\;\;\;\;\;\;\;\;\;\;\;\;\;\;\;if\;\;\;j_{e}>j_{gl}\\ J_{GS}^{e,n}+\chi \left(\frac{J_{GS}^{e,n}-J_{worst}^{n}}{\left(j_{e}-j_{ws}\right)+\varsigma }\right)\;\;\;\;\;\;\;\;\;\;\;\;\;if\;\;\;j_{e}=j_{gl} \end{array}\right\}$$

where $J_{best}^{n}$ is the current global optimal position of the gregarious search agent, θ is the control parameter corresponding to the step size, χ is a random number varying between −1 and 1. $j_{e}$ is the fitness of the present gregarious search agent, $j_{gl}$ is the global best value, and $j_{ws}$ is the global worst solution of the gregarious search agent. Introducing a new parameter based on velocity:

(18) $$J_{best}^{n,\partial }=J_{best}^{n}+V^{n+1}$$

(19) $$J_{best}^{n,\partial }=J_{best}^{n}+\left(V^{n}+\nu_{1}\varpi_{1}\times J_{best}^{n}\right)$$

(20) $$J_{best}^{n,\partial }=J_{best}^{n}\left[1+\nu_{1}\varpi \right]+V^{n}$$

(21) $$J_{GS}^{e,n+1}=J_{best}^{n}+\theta \left|J_{GS}^{e,n}-J_{best}^{n}\left[1+\nu_{1}\varpi \right]+V^{n}\;\right|$$

Finally, the location of search agents depending on the characteristics of corvid and gregarious search agents are hybridized based on [16] as:

(22) $$J_{}^{n+1}=0.5J_{CS}^{n+1}+0.5H_{GS}^{n+1}$$

(23) $$J_{}^{n+1}=0.5[J_{CS}^{e,n}+\lambda_{1}\times a^{e,n}\times \left(Z^{f,n}-J_{CS}^{e,n}\right)]+0.5[J_{best}^{n}+\theta \left|J_{GS}^{e,n}-J_{best}^{n}\left[1+\nu_{1}\varpi \right]+V^{n}\;\right|]$$

(24) $$J_{}^{n+1}=0.5[J_{CS}^{e,n}(1-\lambda_{1}a^{e,n})+\lambda_{1}a^{e,n}\times Z^{f,n}]+0.5[J_{best}^{n}+\theta \left|J_{GS}^{e,n}-J_{best}^{n}\left[1+\nu_{1}\varpi \right]+V^{n}\;\right|]$$

This final Equation (24) is the standard equation comprising the features of corvid and gregarious search agents used in the proposed optimization algorithm.

Step 5: Feasibility check for positions: If the position of the new hybrid seek search agent is feasible, then the location is updated; otherwise, the old position is preserved.

Step 6: Fitness evaluation for a new position: The fitness measure for the entire newly generated hybrid seek search agents is re-evaluated.

Step 7: Update memory for a new position: The memory of the hybrid seek search agents are updated as $J^{n+1}$ when the fitness of the new hybrid seek search agent is better than the fitness of the old hybrid seek search agent.

Step 8: Terminating condition: The above steps are repeated until the termination condition is met. The algorithm involves finding the features of the EEG signal and the hyper-parameters of the ensemble classifier in such a way as to predict the seizure disease with enhanced accuracy. In Algorithm 1, the hybrid seek optimization algorithm’s pseudocode is shown.
**Algorithm 1:** Comprising the features of corvid search and the gregarious search agents.
**Input:**$\;J_{i}^{G}$, i={1,2,…,m} and G={1,2,…,W}
**Output:**
$\;J^{n+1}$
1:   Set and load the population of hybrid seek search agents 2:   Set and initialize maximum iteration, $I_{\max}$, flight length, a, and probability of awareness, $P_{aw}$ 3:   Evaluate fitness function or probability 4:      { 5:     Update the new position relying on Equation (24) 7:     Check for feasibility 8:      Re-evaluate fitness measure 9:       if (*fitness_old_ < fitness_new_*) 10:        { 11:        12:         Replace old solution with new solution 13:        } 14:     Update memory 15:      } 16:     Return $J^{n+1}$ 17:   Terminate

## 4. Performance Evaluation

This section interprets the findings of the proposed seizure prediction module and compares the results to show how well the hybrid seek-based ensemble classifier works in the proposed seizure prediction module. The analysis was carried out using a PYTHON tool that was running Windows version 10 with a 64-bit with 16 GB of RAM OS.

### 4.1. EEG Dataset Description

This research used the standard benchmark data that supports accurate prediction, while the authentication and labeling issues associated with the real-data promoted the use of the datasets mentioned below. In this section, a brief description of the dataset used to test the proposed hybrid seek-based ensemble classifier is provided.

CHB-MIT Scalp EEG Database: This dataset, which is grouped into 23 cases, was obtained from Boston Children’s Hospital and was collected from 22 patients including 5 males (aged 3–22) and 17 females (aged 1.5–19). It is frequently used for the evaluation of latency. The database is composed of pediatric patients’ EEG recordings of persistent seizures. Sixteen bits per second of resolution were used with a sampling rate of 256 samples [29].

Siena Scalp EEG Database: This collection includes information from 14 patients include 9 males (ages 25–71) and 5 females (ages 20–58) admitted to the University of Siena’s department of neurology and neurophysiology. The patients were examined using a Video-EEG at a sampling rate of 512 Hz, with electrodes positioned by the worldwide 10–20 system [30].

### 4.2. Evaluation Metrics

The metrics listed below were used to test the effectiveness of the hybrid seek optimization-ensemble classifier.

Accuracy: Accuracy is formally stated as the rate of closeness between the estimated measure of the system and the actual measures, which is mathematically defined as:

(25) $$Accuracy=\frac{T_{pos}+T_{neg}}{R_{pos}+R_{neg}}$$

Sensitivity: Sensitivity is the probability of the test to result in a genuine positive outcome and is represented by the below equation:

(26) $$Senitivity=\left(\frac{T_{pos}}{no\;\;of\;R_{pos}\;cases}\right)$$

Specificity: Specificity is the probability of the test to result in a genuine negative outcome and is represented by the below equation:

(27) $$Sensitivity=\left(\frac{T_{neg}}{no.\;\;of\;R_{neg}\;cases}\right)$$

### 4.3. Results and Discussion

This section comprises the analytical results from the hybrid seek-based ensemble classifier used to predict a seizure disease. In this section we compare the proposed hybrid seek-based ensemble classifier model of seizure prediction with the most recently reported seizure prediction algorithms.

### 4.4. Comparative Methods

The proposed hybrid seek-based ensemble classifier was compared to other ensemble classifiers, including the AdaBoost classifier (EM1) [31], decision tree classifier (EM2) [6,32], random forest classifier (EM3) [9,33], K-nearest neighbor (EM4) classifier [34], support vector machine (EM5) classifier [13,35], deep learning classifier (EM6) [22], convolutional neural network (EM7) classifier [18,36], crow search optimization-based ensemble classifier [37] (EM8), and squirrel search optimization classifier (EM9) [38].

#### 4.4.1. Analysis Using the CHB-MIT Database

**Figure A1:**
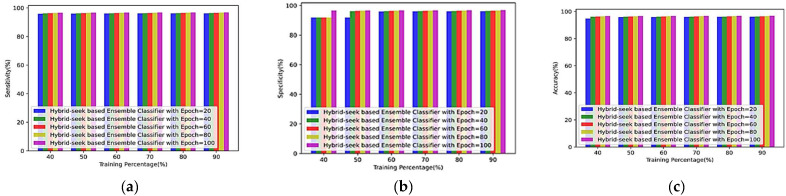
Analysis of performance using CHB-MIT database in terms of training % (**a**) accuracy, (**b**) sensitivity, and (**c**) specificity.

**Figure A2:**
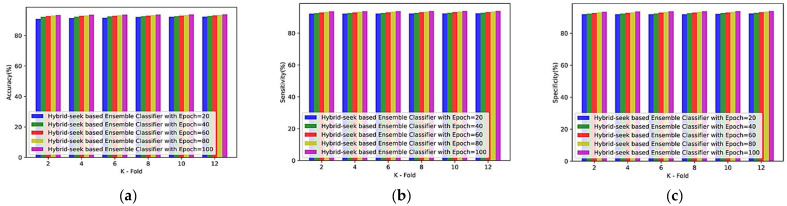
Analysis of performance using the CHB-MIT dataset in terms of k-fold value (**a**) accuracy, (**b**) sensitivity, and (**c**) specificity.

This section discusses the comparative evaluation of the proposed hybrid seek-based ensemble classifier technique using the CHB-MIT dataset in terms of training % and k-fold value. Analysis based on increasing training % and k-fold values on different epoch values is given in Figure A1 and Figure A2 of Appendix A.

(a) Based on the training percentage

**Figure 8:**
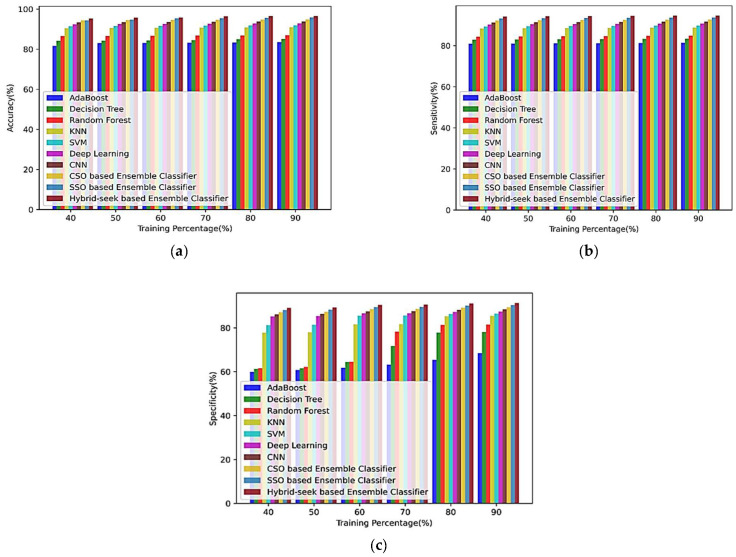
Comparison of the training % of the CHB-MIT database with other classifiers for (**a**) accuracy, (**b**) sensitivity, and (**c**) specificity.

Figure 8 provides an overview of the analysis using the CHB-MIT dataset for training percentages of 40%, 50%, 60%, 80%, and 90% for EM1, EM2, EM3, EM4, EM5, EM6, EM7, EM8, EM9, and the hybrid seek-ensemble classifier. Figure 8a–c shows the comparison of the strategies accuracy, sensitivity, and specificity in terms of the training %.

(b) Based on the k-fold value

**Figure 9:**
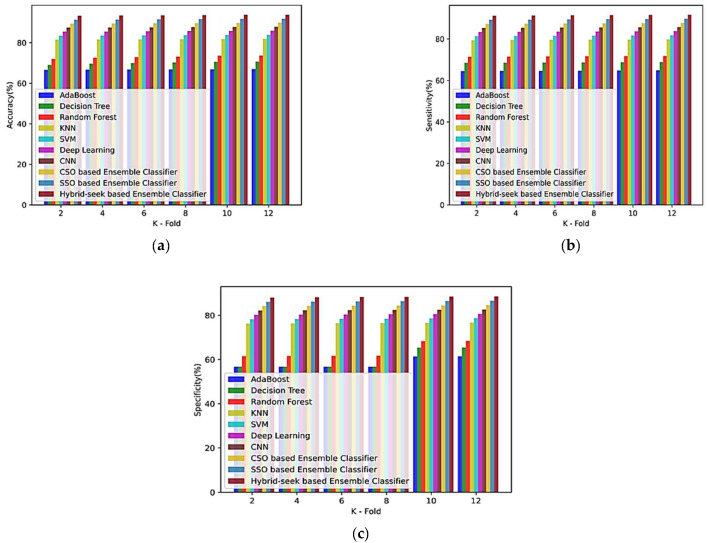
Comparison of k-fold values of the CHB-MIT database with other classifiers for (**a**) accuracy, (**b**) sensitivity, and (**c**) specificity.

The analysis using the CHB-MIT dataset is shown in Figure 9 for the K -fold values of 2, 4, 6, 8, 10, and 12 for EM1, EM2, EM3, EM4, EM5, EM6, EM6, EM7, EM8, EM9, and the hybrid seek-ensemble classifier. Figure 9a–c shows the k-fold measure of accuracy, sensitivity, and specificity of several approaches.

#### 4.4.2. Analysis Using the Siena Scalp Database

**Figure A3:**
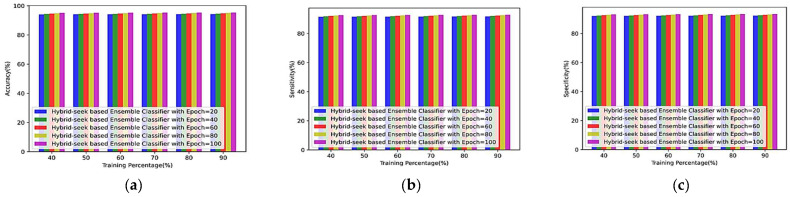
Analysis of performance using Siena database in terms of training % (**a**) accuracy, (**b**) sensitivity, and (**c**) specificity.

**Figure A4:**
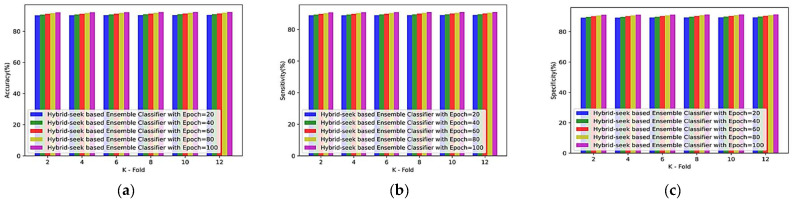
Analysis of performance using the Siena database in terms of k-fold values (**a**) accuracy, (**b**) sensitivity, and (**c**) specificity.

This part compares the training % and k-fold value of a hybrid seek-based ensemble classifier approach on the Siena Scalp dataset. Analysis based on increasing training % and k-fold values on different epoch values is given in Figure A3 and Figure A4 of Appendix A.

(a) Based on the training percentage

**Figure 10:**
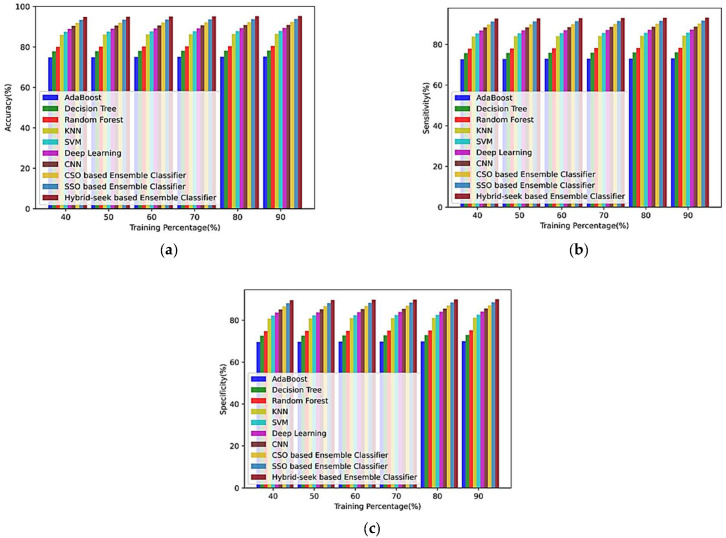
Comparison of the training % of the Siena Scalp database with other classifiers for (**a**) accuracy, (**b**) sensitivity, and (**c**) specificity.

Figure 10 shows the analysis using the Siena dataset for the training percentages based on performance indices such as accuracy, sensitivity, and specificity of the methods, including EM1, EM2, EM3, EM4, EM5, EM6, EM7, EM8, EM9, and the hybrid seek-ensemble classifier. Training percentages of 40%, 50%, 60%, 80%, and 90% are also shown. Figure 10a–c illustrates the proposed approach’s sensitivity, specificity, and accuracy in terms of the training percentage.

(b) Based on the k-fold value

**Figure 11:**
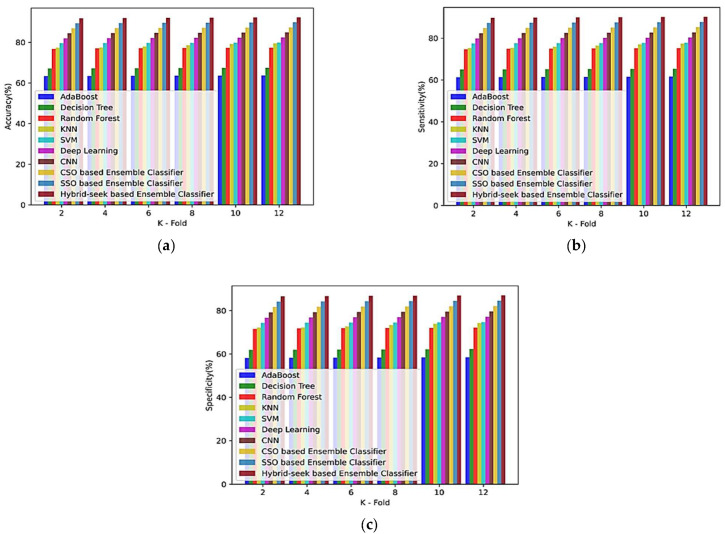
Comparison of k-fold values of Siena Scalp database with other classifiers for (**a**) accuracy, (**b**) sensitivity, and (**c**) specificity.

Figure 11 shows the analysis using the Siena dataset for the k-fold values based on performance indices such as accuracy, sensitivity, and specificity of the methods, including EM1, EM2, EM3, EM4, EM5, EM6, EM7, EM8, EM9, and the hybrid seek-ensemble classifier for the k-fold values of 2, 4, 6, 8, 10, and 12. Figure 11a–c illustrates the approach’s sensitivity, specificity, and accuracy in terms of k-fold values.

### 4.5. Comparative Discussion

In terms of training percentage and k-fold values, Table 2 contrasts several performance indices based on different approaches using the CHB-MIT dataset. Regarding training percentage, the hybrid seek-based ensemble classifier’s accuracy, sensitivity, and specificity are 96.6120%, 94.6736%, and 91.3684%, respectively. Similar to this, the accuracy, sensitivity, and specificity of the hybrid seek-based ensemble classifier in terms of the k-fold value are 93.812%, 93.812%, and 88.5684%, respectively.

In terms of training percentage and k-fold value, a comparative analysis of different approaches based on accuracy, sensitivity, and specificity using the Siena Scalp dataset is shown in Table 3. Table 3 shows that the accuracy, sensitivity, and specificity of the proposed hybrid seek-based ensemble classifier in terms of training percentage are 95.3090%, 93.1766%, and 90.0654%, respectively. Similarly, the accuracy, sensitivity, and specificity of the hybrid seek-based ensemble classifier in terms of k-fold value are 92.3150%, 90.1826%, and 87.0714%, respectively.

As a result, it is clear that the hybrid seek-based ensemble classifier, compared to comparable approaches, can offer superior seizure prediction while achieving higher accuracy, sensitivity, and specificity measures.

## 5. Conclusions and Future Scope

This paper proposes an ensemble classifier with an optimization-based optimization module for automated seizure prediction. Pre-processing is first applied to the EEG data set to eliminate any noise that may be present. The significant statistical, wavelet-based, and entropy-based features are then retrieved from the alpha, beta, delta, theta, and gamma waves of the EEG data. The features are extracted with the proposed hybrid seek algorithm and developed with the corvid and gregarious search agents. The features that have been successfully extracted are then given to the ensemble classifier, consisting of the AdaBoost, random forest, and decision tree classifiers. The fusion parameters are evaluated using the proposed hybrid seek optimization algorithm to provide precise seizure prediction at an early stage. The accuracy, sensitivity, and specificity performance indices were used to evaluate the performance of the suggested method, and they were found to be 96.6120%, 94.6736%, and 91.3684%, respectively, for the CHB-MIT database, and 95.3090%, 93.1766, and 90.0654%, respectively, for the Siena Scalp dataset. These values are high compared to other methods, and we are confident that this will help in information processing for complex medical diseases, such as seizures, to improve management of these diseases in the future. COVID-19-affected people have a high chance of developing seizures; therefore, in the future, if we analyze the data of COVID-19-affected people, then seizure prediction could be performed more efficiently.

## Figures and Tables

**Table 1:** Representation of feature vectors.

Number of Samples/Feature Vectors	0	1	2	…	128	129
0	−4.78 × 10^−16^	2.06 × 10^−27^	4.54 × 10^−14^	…	0.34987	8.13 × 10^−28^
1	1.35 × 10^−16^	5.15 × 10^−28^	2.27 × 10^−14^	…	0.34677	2.90 × 10^−28^
2	−7.42 × 10^−17^	3.13 × 10^−28^	1.77 × 10^−14^	…	0.33928	2.26 × 10^−28^
3	−1.69 × 10^−17^	1.88 × 10^−27^	4.33 × 10^−14^	…	0.33216	7.34 × 10^−28^
4	8.69 × 10^−17^	1.63 × 10^−27^	4.03 × 10^−14^	…	0.4069	7.40 × 10^−28^
5	2.93 × 10^−16^	1.67 × 10^−27^	4.08 × 10^−14^	…	0.31926	8.24 × 10^−28^
6	−5.95 × 10^−16^	1.56 × 10^−27^	3.95 × 10^−14^	…	0.32999	6.32 × 10^−28^
7	−5.78 × 10^−16^	1.76 × 10^−27^	4.19 × 10^−14^	…	0.3514	1.19 × 10^−27^
8	−2.55 × 10^−17^	3.42 × 10^−27^	5.85 × 10^−14^	…	0.3786	1.41 × 10^−27^
9	−1.77 × 10^−16^	1.02 × 10^−27^	3.19 × 10^−14^	…	0.38145	6.72 × 10^−28^

**Table 2:** Comparison of methods using the CHB-MIT database.

Methods	Training Percentage			k-Fold Value		
	Accuracy (%)	Sensitivity (%)	Specificity (%)	Accuracy (%)	Sensitivity (%)	Specificity (%)
Adaboost	83.4833	81.3509	68.4699	66.9866	64.8542	61.4750
Decision Tree	85.0614	83.3334	78.0727	70.6876	68.8392	65.4440
Random Forest	86.9478	84.8154	81.5302	73.6596	71.8232	68.4160
K-NN	90.9272	88.7948	85.5016	81.9344	79.8020	76.6908
SVM	91.9080	89.7756	86.4804	83.9160	81.7836	78.6724
Deep Learning	92.8884	90.7560	87.4588	85.8968	83.7644	80.6532
CNN	93.8684	91.7360	88.4368	87.8768	85.7444	82.6332
CSO-based classifier	94.8480	92.7156	89.4144	89.8560	87.7236	84.6124
SSO-based classifier	95.8272	93.6948	90.3916	91.8344	89.7020	86.5908
Proposed Hybrid Seek-based Ensemble Classifier	96.6120	94.6736	91.3684	93.8120	91.6796	88.5684

**Table 3:** Comparison of methods using the Siena Scalp database.

Methods	Training Percentage			k-Fold Value		
	Accuracy (%)	Sensitivity (%)	Specificity (%)	Accuracy (%)	Sensitivity (%)	Specificity (%)
Adaboost	75.2349	73.1025	69.9914	63.7245	61.5921	58.4809
Decision Tree	78.2187	76.0863	72.9751	67.4595	65.3271	62.2159
Random Forest	80.4517	78.3193	75.2081	77.3605	75.2281	72.1169
K-NN	86.4308	84.2984	81.1872	79.5200	77.3876	74.2764
SVM	87.9120	85.7796	82.6684	79.9200	77.7876	74.6764
Deep Learning	89.3926	87.2602	84.1490	82.4010	80.2686	77.1574
CNN	90.8726	88.7402	85.6290	84.8810	82.7486	79.6374
CSO-based classifier	92.3520	90.2196	87.1084	87.3600	85.2276	82.1164
SSO-based classifier	93.8308	91.6984	88.5872	89.8380	87.7056	84.5944
Proposed Hybrid Seek-based ensemble classifier	95.3090	93.1766	90.0654	92.3150	90.1826	87.0714

## Data Availability

The data used for this paper are cited within the article.

## Appendix A

This additional section details the analytical results from the hybrid seek-based ensemble classifier used to predict seizure diseases using CHB-MIT and Siena Scalp databases based on training percentage and k-fold values.

(a) Analysis using the CHB-MIT database: This section evaluates the training percentage and k-fold values of a hybrid seek-based ensemble classifier using the CHB-MIT dataset.

(i) Based on the training percentage: Figure A1 illustrates the analysis using the CHB-MIT dataset for the training percentage based on performance indices such as accuracy, sensitivity, and specificity. These parameters of the hybrid seek-based ensemble classifier are calculated at an epoch of 20, 40, 60, 80, and 100 in terms of training percentages of 40%, 50%, 60%, 80%, and 90%.

(ii) Based on the k-fold value: Figure A2 illustrates the analysis using the CHB-MIT dataset for k-fold values based on performance indices such as accuracy, sensitivity, and specificity. These parameters of the hybrid seek-based ensemble classifier are calculated at an epoch of 20, 40, 60, 80, and 100 in terms of k-fold values of 2, 4, 6, 8, 10, and 12.

(b) Analysis using Siena Scalp database: The analysis of the hybrid seek-based ensemble classifier with the Siena Scalp dataset in terms of training percentage and k-fold value is detailed in this section.

(i) Based on the training percentage: Figure A3 illustrates the analysis using the Siena dataset for the training percentage based on performance indices such as accuracy, sensitivity, and specificity. These parameters of the hybrid seek-based ensemble classifier are calculated at the epoch of 20, 40, 60, 80, and 100 in terms of training percentages of 40%, 50%, 60%, 80%, and 90%.

(ii) Based on the k-fold value: Figure A4 illustrates the analysis using the Siena dataset for k-fold values based on performance indices such as accuracy, sensitivity, and specificity. These parameters of the hybrid seek-based ensemble classifier are calculated at an epoch of 20, 40, 60, 80, and 100 in terms of k-fold values of 2, 4, 6, 8, 10, and 12.
